# False-Positive Cardiac Troponin Elevations in Skeletal Muscle Disease: Clinical Relevance, Mechanisms, and Laboratory Approaches

**DOI:** 10.33549/physiolres.935683

**Published:** 2025-10-01

**Authors:** Daniel RAJDL, Marie ŠOLCOVÁ, Jaroslav RACEK, David SUCHÝ, Hana VIMMEROVÁ, Pavel BROZ, Pavel PROKOP

**Affiliations:** 1 Department of Clinical Biochemistry and Hematology, University Hospital in Pilsen and Faculty of Medicine in Pilsen, Charles University, Pilsen, Czech Republic; 2 Department of Clinical Pharmacology, University Hospital in Pilsen, Pilsen, Czech Republic

**Keywords:** Cardiac troponin, Skeletal muscle disease, False-positive results, Analytical interference, Macrotroponin, Laboratory diagnostics

## Abstract

Cardiac troponins are indispensable biomarkers for the diagnosis of acute myocardial infarction, but false-positive elevations that contradict the clinical picture remain a significant challenge in laboratory medicine. Analytical interferences may arise from macrotroponins, heterophile or anti-troponin antibodies, and the limited cardiac specificity of high-sensitivity troponin T (hs-cTnT) in skeletal muscle disease. Recent studies show that hs-cTnT is elevated in up to two-thirds of patients with myopathies, while high-sensitivity troponin I (hs-cTnI) is largely unaffected, underscoring the diagnostic advantage of hs-cTnI in this setting. A pragmatic diagnostic approach should combine clinical plausibility with stepwise laboratory testing. First, preanalytical factors such as sample mislabeling, fibrin clots, or hemolysis must be excluded and the measurement repeated. If the results remain incongruent, an alternative assay – ideally hs-cTnI – should be performed. Further evaluation may include heterophile-blocking reagents, polyethylene glycol precipitation to screen for macro-analytes, and, where available, confirmatory techniques such as gel filtration chromatography or immunoglobulin depletion (protein A/G). Although these strategies can help identify assay interference, there is no universally accepted gold standard. Awareness of false-positive elevations, careful interpretation of discordant troponin results, and effective collaboration between laboratories and clinicians are essential to prevent misdiagnosis and unnecessary interventions. Clear documentation of confirmed interferences further ensures safe patient management and provides guidance on which assays are reliable for future tests.

Cardiac troponins (cTn) are the cornerstone biomarkers for the diagnosis of acute myocardial infarction (AMI), particularly in the context of suspected acute coronary syndrome (ACS). According to current guidelines [1,2], a dynamic rise and/or fall of troponin concentrations with at least one value above the 99^th^ percentile upper reference limit (URL), in conjunction with clinical evidence of myocardial ischemia, defines myocardial infarction. Elevated cTn levels are also observed in myocarditis, toxic cardiomyopathy, Tako-Tsubo syndrome, heart failure, pulmonary embolism, or critical illness, reflecting both the utility and limited specificity of these biomarkers.

In clinical practice, troponin elevations can occasionally be inconsistent with the patient’s status. Analytical interference should then be considered. Documented causes include macrotroponins (up to 5 % of samples in some assays [3]), heterophile antibodies, rheumatoid factor, and assay-specific cross-reactivity. A particularly challenging and underrecognized scenario is the elevation of cardiac troponin T (cTnT) in patients with skeletal muscle diseases, in whom the cardiac origin of troponin may be falsely inferred.

A 27-year-old woman was admitted with severe rhabdomyolysis (CK>360 μkat/l, myoglobin >5000 μg/l) and showed markedly elevated hs-cTnT (73 ng/l; URL 9 ng/l) but normal ECG and echocardiography. Workup with heterophile-blocking reagents and polyethylene glycol precipitation did not confirm antibody or macrocomplex interference. The patient fully recovered after corticosteroid therapy and no cardiac pathology was demonstrated. This case illustrates the diagnostic challenge of interpreting troponin results in skeletal muscle disease.

A typical indicator of possible analytical interference or lack of specificity is persistently elevated troponin without dynamics and without clear clinical correlation. In addition to false-positive elevations of hs-cTnT in muscle damage, the two most common interferences include the presence of heterophilic antibodies (e.g., human anti-mouse antibodies, HAMA) or the presence of macrotroponins. Such results may trigger unnecessary investigations. Awareness of these pitfalls and close communication between laboratories and clinicians are crucial.

Practical evaluation begins with repeat testing and exclusion of preanalytical problems such as mislabeling, fibrin clots, or hemolysis. If the results remain discordant, measurement with an alternative assay should be performed, preferably hs-cTnI. Further testing may include heterophile-blocking reagents, PEG precipitation, and confirmatory methods such as size-exclusion chromatography or immunoglobulin depletion. A stepwise approach is summarized in Figure 1.

In patients with skeletal muscle diseases, elevations in hs-cTnT are frequent (45–69 %) while hs-cTnI is usually unaffected [4–6]. For example, hs-cTnT elevations were reported in 69 % of hereditary or acquired myopathies [7], 46 % of idiopathic inflammatory myopathies [8], and 55 % of chronic skeletal muscle disease [9], with much lower prevalence for hs-cTnI. Table 1 summarizes recent studies. Strong correlations with CK and myoglobin support a skeletal rather than cardiac origin. The mechanisms likely include re-expression of cTnT in diseased muscle and cross-reactivity of assay antibodies [9–12].

Macrotroponins are troponin complexes with immunoglobulins (mainly IgG). The mechanism of false-positive results caused by this interference is often explained mechanistically as with other macromolecules (e.g. macroamylase) by prolonging the biological half-life. However, this notion does not explain the frequent observation that macrotroponins interfere only with some analytical kits [3], this interference is described in the literature even for variants of the analysis kit from the same manufacturer [13], and our experience that different types of biological materials (serum vs. plasma) give significantly different results (unpublished data). Macrotroponins are relatively (compared to HAMA anti-bodies) more common causes of spurious long-term increases in troponin concentrations and are therefore essential to keep in mind in obscure clinical conditions with persistently elevated troponin. It is likely that in some populations, e.g. athletes who have had a COVID-19 infection, the prevalence of macrotroponin is very high (88 %) and leads to falsely high results in some manufacturers’ measurement kits [14]. The recently published case series and a review of the literature confirm that macrotroponin is a relatively frequent cause of unexplained persistent troponin elevation, underscoring the importance of recognizing this analytical interference in clinical practice [15].

Polyethylene glycol (PEG; eg, PEG 6000) precipitation is a useful technique to detect macro-analytes and identify immunoglobulin-related interferences in immunoassays, as recommended by Fahie-Wilson [16]. However, PEG precipitation is a nonspecific method that separates proteins on the basis of solubility and may precipitate a variable proportion of free analyte depending on the substance. Due to its lack of specificity and the potential to interfere with some immunoassays, it is recommended to establish method- and analyte-specific reference ranges and interpret results with caution. Although the reference range for percentage recovery is often highly variable for different analytes [17] and can also be affected by the concentration of the free analyte, generally the results of PEG precipitated samples lower than 20 % of original values are considered “positive” [4]. Although PEG precipitation is commonly used for macroprolactin or macroamylase screening, it should not be used as a universal screening tool for all analytes. Positive results should be confirmed by more specific gel filtration. Gel filtration chromatography (size-exclusion chromatography) separates molecules on the basis of their molecular weight and size. The macrocomplexes of troponin have a significantly higher molecular weight than free troponin or typical immunoglobulins (such as IgG). The presence of cTn immunoreactivity in fractions with higher molecular weight supports the presence of macrotroponin. The procedure is typically performed on columns (e.g., Sephacryl S-300HR or Sephadex G100) using a buffered saline solution as the eluent, with cTn concentrations measured in individual fractions. Gel filtration is an analytically specific and robust method that allows direct visualization of both bound and unbound troponin. However, it is not available in real time, requires multiple troponin measurements, and requires a high level of laboratory expertise.

Heterophilic antibodies are rather rare, but the exact prevalence is unknown. The most common causes are viral diseases, contact with animals (especially rodents), vaccination, autoimmune diseases (e.g., presence of rheumatoid factor) or the use of certain drugs. To confirm the presence of heterophilic antibodies, we most often use the heterophilic blocking tube (HBT; Scantibodies Laboratory, Inc., Santee, California, USA, Part Number: 3IX762). The tube contains a lyophilized mixture of animal immunoglobulins to counterbalance heterophilic antibodies. The principle of this test is straightforward: patient plasma is incubated with a mixture of animal immunoglobulins that neutralize heterophilic antibodies, and troponin is then remeasured to detect any change in concentration. Mechanistically, HAMA can bind to the murine components of the capture and/or detection anti-bodies used in the assay. This binding may generate a signal even in the absence of troponin, resulting in a false-positive result, or block epitope binding and lead to a false-negative result.

Other analytical principles less used to detect immunoglobulin interference are immunoglobulin depletion by protein A/G [18] or serological tests that detect antibodies to troponin [6]. Proteins A and G are bacterial proteins with high binding affinity to immunoglobulins, especially IgG. Protein A binds primarily to IgG, while protein G interacts with a wider range of IgG subclasses and, in some cases, other immunoglobulin classes, making it a more universal tool. In depletion experiments, the plasma or serum of the patient is incubated with a resin containing protein A and/or G (commonly in the form of spin columns or beads). During incubation, antibodies, including those that form macrocomplexes with analytes such as troponin, bind to proteins A and G and are subsequently removed from the sample by centrifugation. Then, the analyte concentration is measured in the supernatant. This method is considered analytically specific for the removal of immunoglobulins. Despite the availability of a wide range of methods, there is no gold standard for the detection of false-positive elevations.

## Figures and Tables

**Fig. 1:**
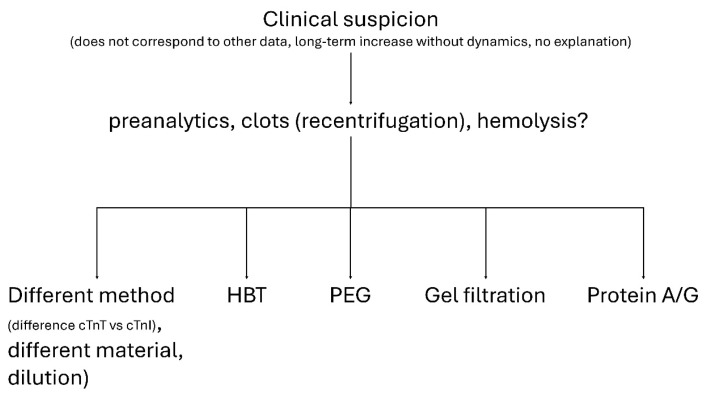
Algorithm for the detection of false-positive cardiac troponin results. Flow chart illustrating the recommended laboratory approach to investigate suspected false-positive troponin results. After preanalytical issues are excluded, further steps include alternative methods (cTnT vs. cTnI), HBT, PEG precipitation, gel filtration, and immunoglobulin depletion using protein A/G to identify assay interference or macrocomplexes. In the case of cTnT, possible false-positive elevations should always be considered in muscle diseases.

**Table 1:** Summary of recent studies reporting cTnT results in patients with myopathies.

Study	Patients (n)	Population	% elevated hs-cTnT (>14 ng/l)	Median hs-cTnT (IQR)	% Elevated hs-cTnI (cutoff)
*Schmid et al. [7]*	74	Hereditary & acquired myopathies	68.9 %	24 (11–54) ng/l	4.1 % (>26 ng/l)
*Barros E Silva et al. [8]*	61	Idiopathic inflammatory myopathies	45.9 % 6.2 % in controls	12.0 (5.0–35.0) ng/l 5.0 (4.0–6.2) ng/l in controls	Not directly reported, but hs-cTnI is less frequently abnormal and not significantly different from controls.
*du Fay de Lavallaz et al. [9]*	211	Chronic active skeletal muscle disease	55 %	16 (7–32.5) ng/l vs. 5 (3–9 ng/l) in controls	8–23 % (depending on the hs-cTnI assay)
